# Exploring the use of metacognitive monitoring cues following a diagram completion intervention

**DOI:** 10.1007/s10459-023-10309-9

**Published:** 2024-01-29

**Authors:** Babu Noushad, Pascal W. M. Van Gerven, Anique B. H. de Bruin

**Affiliations:** 1 Department of Educational Development and Research, School of Health Professions Education (SHE), Maastricht University, P.O. Box 616, 6200 MD Maastricht, The Netherlands; 2 College of Health Sciences, University of Buraimi, P.O. Box 890, PC 512 Al Buraimi, Sultanate of Oman

**Keywords:** Monitoring, Metacognition, Predictive cues, Diagrams, Think-aloud

## Abstract

Studying texts constitutes a significant part of student learning in health professions education. Key to learning from text is the ability to effectively monitor one’s own cognitive performance and take appropriate regulatory steps for improvement. Inferential cues generated during a learning experience typically guide this monitoring process. It has been shown that interventions to assist learners in using comprehension cues improve their monitoring accuracy. One such intervention is having learners to complete a diagram. Little is known, however, about how learners use cues to shape their monitoring judgments. In addition, previous research has not examined the difference in cue use between categories of learners, such as good and poor monitors. This study explored the types and patterns of cues used by participants after being subjected to a diagram completion task prior to their prediction of performance (PoP). Participants’ thought processes were studied by means of a think-aloud method during diagram completion and the subsequent PoP. Results suggest that relying on comprehension-specific cues may lead to a better PoP. Poor monitors relied on multiple cue types and failed to use available cues appropriately. They gave more incorrect responses and made commission errors in the diagram, which likely led to their overconfidence. Good monitors, on the other hand, utilized cues that are predictive of learning from the diagram completion task and seemed to have relied on comprehension cues for their PoP. However, they tended to be cautious in their judgement, which probably made them underestimate themselves. These observations contribute to the current understanding of the use and effectiveness of diagram completion as a cue-prompt intervention and provide direction for future research in enhancing monitoring accuracy.

## Introduction

A significant portion of a student’s learning relies on text-based materials. This includes tasks such as reading a case report to complete a clinical assignment, preparing for an upcoming exam and summarizing research. Comprehension, or the process of deeply understanding a text and integrating its ideas with prior knowledge, is crucial for learning (Kintsch, 1998). If students are given a text about the process of visual signal transfer through the optic nerve, the one with good comprehension ability should capture, recreate, and explain the visual signal transfer process described in the text. Research has shown that students with better comprehension skills tend to perform well academically (Akbasli et al., 2016). Metacomprehension, or the ability to evaluate one’s own comprehension, is important for regulating learning efforts (Wiley et al., 2005). This helps learners distinguish between texts that they have well understood and those that they have not. Accurate monitoring of learning is essential for regulating learning and performance, as it helps steer learning efforts towards areas that require further improvement (Dunlosky & Lipko, 2007; Nelson & Narens, 1990). For instance, being able to assess whether they have fully mastered a topic, if they need additional practice to improve their skills, or if they need more exposure to a particular specialty would allow students to allocate their time and efforts more efficiently (Thiede et al., 2003).

The monitoring accuracy of learners has been studied by comparing their perceived performance in a task with their actual performance (Redford et al., 2012; Thiede et al., 2009, 2022; van Loon et al., 2014). The results suggest that learners often find it difficult to judge their performance accurately. The findings from a recent meta-analysis of 94 empirical studies suggest that learners have a moderate ability to distinguish between texts that are comprehended well and those that are not (*relative metacomprehension accuracy* = 0.24) (Prinz et al., 2020a). In healthcare, physicians’ self-assessment capabilities and subsequent self-regulated learning efforts are closely linked to the quality of care they provide (Murdoch‐Eaton & Whittle, 2012; Sandars & Cleary, 2011). However, the evidence demonstrates a weak correlation between physicians’ self-rated assessments and standardized external assessments (Davis et al., 2006; Eva et al., 2004). Among health profession trainees, the correlation between self and external assessments of knowledge has been shown to range between 0.02 and 0.65 (Gordon, 1991). Thiede and colleagues (2010) believe that such disparity in self-monitoring occurs because learners fail to recognize and utilize the most informative mental indicators (known as ‘cues’) that guides monitoring decisions (Thiede et al., 2010). This emphasizes the need to enhance the monitoring accuracy of health professionals as early as during their education and training (de Bruin et al., 2017). Prompting students to utilize cues that are more indicative of their level of learning has proven to be an effective intervention for enhancing monitoring accuracy and thereby promoting self-regulated learning (Anderson & Thiede, 2008; de Bruin et al., 2011; Griffin et al., 2008; Redford et al., 2012; Thiede et al., 2010). One such approach is completing diagrams of causal relations described in a learning content (van Loon et al., 2014). It may assist learners in retrieving valid and relevant information from memory and, in the process, provides cues that can predict their level of comprehension. Recent research utilizing diagram completion as a cue-generating activity has demonstrated enhanced monitoring accuracy (Van de Pol et al., 2019; van Loon et al., 2014). Yet, it remains unclear from this research what specific cues learners utilize in making their monitoring decisions, and how the use of these cues varies among individuals. The current study aims to narrow this gap by studying the underlying mental processes while learners engage in a diagram completion task and subsequently evaluate their level of learning.

## Improving monitoring accuracy

The quality of monitoring judgments—that is, the monitoring accuracy—is based on how closely learners can predict their actual performance in a forthcoming evaluation or assessment. This monitoring judgement is complex because learners lack direct access to the quality of their cognitive states (Koriat, 1997). When students learn a concept, they cannot directly check how well it is represented in their memory to estimate how well they will recall it in a future test. In such situations, they must base their judgment on ‘cues’ they gather while monitoring their learning. The precision of monitoring judgments therefore relies on the cues that learners prefer to use. Higher accuracy is obtained with cues tied to the mental representation of the text content, which determines the performance of subsequent test results (Prinz et al., 2020a). Utilizing cues representative of text comprehension such as “my ability to explain the text content” will result in better accuracy than using cues such as “my familiarity with the subject” or “easier processing” of the text content.

Monitoring accuracy can be expressed in several ways, including “absolute accuracy”, “relative accuracy”, or “bias index” (Burson et al., 2006; Nelson, 1996). *Absolute accuracy* is measured as the difference between the predicted (judgement) and actual performance scores. Therefore, it measures the judgment precision as an absolute value. If this difference is smaller (i.e., closer to zero), the absolute accuracy is higher. The *bias index* is another form of absolute accuracy that measures the extent to which an individual is overconfident or underconfident while making a prediction. The *bias index* can be positive or negative, indicating the direction and amount of discrepancy between judgement and performance. A positive value denotes overconfidence, whereas a negative value denotes underconfidence (Schraw, 2009). *Relative accuracy* indicates how well a student is able to discriminate what they have learned or performed on one task compared to another. This measure captures the extent to which learners can judge whether understanding or performance is higher, lower, or similar when comparing between tasks. In real world learning settings, this aspect of metacognitive monitoring is important when deciding what (parts of) tasks need priority or no longer need attention in further regulation of learning. The relative accuracy is usually represented as a *gamma* correlation, which indicates the strength of the association between judgment and test performance. Its values range from + 1 (perfect positive association) to − 1 (perfect negative association) and a value of ‘zero’ signifies the absence of any association between the judgment and the test performance. An elaboration of these monitoring accuracy measures and their relevance to learning contexts are outlined in Table 1.

**Table 1:**
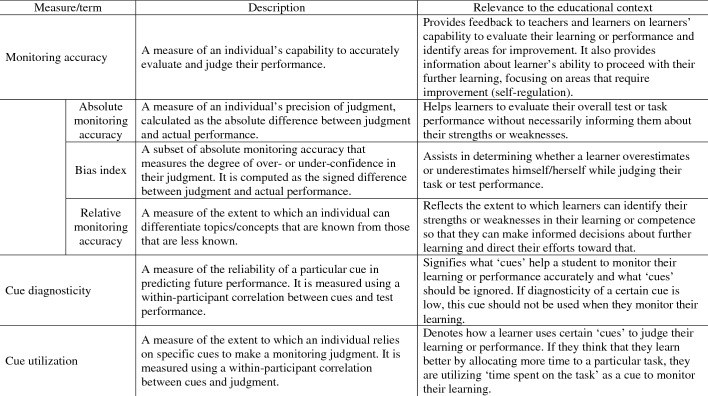
Description of monitoring accuracy measures and their relevance to learning context

According to the construction-integration model of text comprehension (Kintsch, 1998), a learner simultaneously constructs mental representation of texts at multiple levels. First, a surface level is built while the words are being encoded. Second, a text-based level is built as the words are broken down into propositions and connections are made between these propositions. Finally, a situation-model level is constructed as various textual components are linked to one another and to the learner's existing knowledge. The situation model exemplifies the learner's deeper understanding of the text. Several interventions have been developed based on the construction-integration model to aid students in utilizing more appropriate cues to enhance their monitoring accuracy. As a part of these experiments, learners were asked to read expository text materials. This was followed by interventions such as writing a summary of the text (Anderson & Thiede, 2008) (*M* Gamma = 0.64), preparing keywords that represent the gist of the text content (de Bruin et al., 2011) (*M* Gamma = 0.27), self-explaining the meaning of content in the text (Griffin et al., 2019) (*M* Gamma = 0.46), or preparing a visual representation of the connection between concepts in a text (Redford et al., 2012) (*M* Gamma = 0.41). These interventions helped learners to generate, access, or select situation-model cues (Prinz et al., 2020a). Following the cue-generation interventions, learners were asked to predict their performance (PoP) in a criterion test at the end of the experiment. Monitoring accuracy was then obtained by correlating their PoP with their actual test score in the criterion test. This monitoring accuracy was observed to be greater if the cue-generation task was performed after a delay than if it was performed immediately. The delayed cue generation tasks are assumed to help learners to disregard the surface and text-level mental representation of text content in favor of situation-level comprehension because the former have decayed from working memory after the delay (Anderson & Thiede, 2008; van Loon et al., 2014).

## Diagram completion—a generative cue intervention

Diagrams provided alongside textual content are known to improve students’ metacomprehension (Butcher, 2006). Van Loon and colleagues (2014) and Van de Pol and colleagues (2019) investigated the usefulness of asking learners to complete a pre-structured empty diagram illustrating the cause-effect relationships between the concepts given in a text. In these experiments, learners attempted to portray the sequential steps of a causal chain from the studied text. To accomplish the diagram completion task, learners were required to identify the key concepts from the studied text and infer their causal relationships. Engaging in the diagram completion task is therefore expected to provide learners with more valid cues predictive of their learning because of the visual and comprehensive nature of the task. Van Loon and colleagues (2014) observed an increase in relative monitoring accuracy (*M* Gamma = 0.56) after a delayed completion of diagrams and reasoned that learners based their judgment on cues that indicated an actual understanding of the causal connections in the texts. Van de Pol et al., (2019, Exp. 1) observed a higher monitoring accuracy (*M* Gamma = 0.43) for causal relations among secondary school students. According to them, cues such as the number of correct causal relations, omissions in the diagrams, and the number of completed text boxes were most predictive of participants’ test performance.

Learners use a variety of cues to monitor their learning and to make predictions of their future performance. The accuracy of their prediction depends upon the quality of these cues. This is known as *cue diagnosticity*. Thus, cue diagnosticity is the level of a cue’s ability to predict a future performance. However, learners might vary in utilizing these cues. *Cue utilization*, therefore, describes the degree to which these cues have been utilized when learners make their judgment (Van de Pol et al., 2019; van Loon et al., 2014). Van Loon et al. (2014) observed that the diagram cues such as ‘correct causal relationship’ and the ‘number of omitted text boxes’ were predictive of future test performance (cue diagnosticity). For instance, the correlation between the correct causal relationship and the test performance was highly positive (0.53), indicating that providing a correct causal relationship in the diagram was strongly related to having a higher test score. The participants in that study seemed to have utilized the same set of cues—‘correct causal relationship’ and ‘omission errors’—to support their PoP (cue utilization). They also observed that the correlation between omission errors and PoPs was negative (− 0.64) indicating that the participants provided lower PoPs when their diagram text boxes contained more unfilled text boxes. Similarly, a diagram completion intervention among secondary school students has revealed cues such as the number of correct causal relations, the number of omissions present in the diagram, and the number of boxes completed were significantly correlated with students’ test scores (cue diagnosticity) and the participants used similar sets of cues for their PoP (cue utilization) (Van de Pol et al., 2019). These observations suggest that diagram completion activity generates predictive cues.

Despite the fact that engaging in a diagram completion task provides learners with cues indicative of their level of comprehension, how learners select, interpret, and apply these cues may vary. Previous diagramming studies have tried to establish cue use in a more ad hoc and correlational manner (Van de Pol et al., 2019; van Loon et al., 2014). Although these studies were able to ascertain which cues obtained from the student’s diagrams were diagnostic, they were unable to identify which cues students focused on when making monitoring decisions. In addition, they did not examine the variation in cue use between categories of learners, such as good and poor monitors. For instance, diagramming studies reveal that correct text boxes or omitted text boxes are cues that are diagnostic. However, the accessibility and use of these cues may differ between good and poor monitors. This raises the question of how the monitoring practices of individuals differ, which potentially has implications for the design of adaptive interventions to improve their monitoring accuracy.

This is the first attempt at exploring a diagram intervention and its related issues of cue use in the health professions education context. In order to promote self-regulated learning, medical educators are incorporating instructional approaches to improve students' metacognitive skills. Given that accurate self-monitoring is a necessity for self-regulated learning, an in-depth understanding of the learner's monitoring process in such contexts is crucial, particularly in light of the literature indicating variation in self-assessment accuracy among physicians (Davis et al., 2006; Johnson et al., 2023). Therefore, gaining a deeper understanding of participants’ cue use through a more direct observation of cognitive processes during cue use might help our attempts to improve the monitoring accuracy of learners.

## The present study

In order to guide the efforts to enhance the monitoring accuracy of students' comprehension of complex texts, it is important to widen our understanding of learners’ cue use patterns. The present study, therefore, set out to answering the following research questions:

1. What cues do learners utilize to base their learning monitoring on after a diagram completion activity illustrating causal relationships?
2. How does the pattern of cue use differ between good and poor monitors of learning?

A set of novel and complex expository texts were developed from an undergraduate optometry curriculum and piloted to choose six texts for the experiment. Participants read these texts sequentially, completed diagrams illustrating the causal relationships in the texts, and predicted how well they would perform on a test designed to assess their comprehension of cause-and-effect relationships. The difference between the predicted and actual performance scores were estimated to determine the monitoring accuracy (i.e., absolute accuracy) and used to categorize the participants as either good or poor monitors. They spoke aloud their thought processes while completing the diagram and during their PoP. These verbal accounts were subjected to a reflexive thematic analysis to determine the types and patterns of cues used by good and poor monitors. This study did not further explore absolute monitoring accuracy, other than employing it to classify learners, and only reported the relative monitoring accuracy to maintain consistency with previous diagramming research. Similarly, the study did not estimate the ‘cue diagnosticity’ or the ‘cue utilisation’ of participants as they were outside the scope of this work.

## Methodology

### Philosophical stance and researcher positioning

This study employs nested mixed methods research with a qualitative focus and a quantitative component. The researchers adopt a pragmatic philosophical stance in which they assert that the interweaving of qualitative and quantitative methodologies provides a logical grounding, methodological flexibility, and an in-depth understanding of the research questions. In this study, the monitoring accuracy of the participants following the cue-generative diagram completion intervention is estimated quantitatively, and the cue use that guides their judgement of learning is explored qualitatively. BN is an experienced educator in the health professions who is interested in studying the cognitive processes of student learning. ABHdB is a professor of educational psychology, focusing on understanding how to support students’ self-regulation of learning in higher education. PWMVG is an experimental psychologist who has a broad interest in cognitive functions and their role in learning. These backgrounds aided us in the co-construction of interpretations while resolving our research questions, which seek to translate cognitive and educational psychology works into medical education.

### Study context and participants

The data was collected between September 2020 and April 2021 from 31 undergraduate optometry students (27 females, 4 males) at the College of Health Sciences, University of Buraimi, Sultanate of Oman. Students in their second professional year were chosen for this study on the assumption that they would have acquired the appropriate background knowledge to comprehend novel concepts of primary eye care. Participants were identified based on voluntary participation and verbal ability. Each participant's verbal proficiency was determined by requesting input from their respective course instructors. Thereafter potential candidates were contacted by email and invited to join. BN is a senior faculty member at the same organization who recruited the participants but has had no teaching or administrative interactions with them in the past or during the course of the study. The research and ethics committee at the College of Health Sciences, University of Buraimi (Ref No:0030/REC/2019), approved this study.

### Study instrument: expository text materials

Expository texts provide detailed, organized information on a subject. Thirteen texts describing novel and complex concepts from the optometry curriculum were prepared so that the logical organization of the content led to serial or simultaneous causal relationships (see Appendix A & B for sample texts and diagrams). Each text had five distinct idea units in the causal chain. Hence, five text boxes were included in the diagram that followed the texts. All the text materials were prepared in a single paragraph format with a mean (± *SD*) word count of 198 ± 28.

A pilot study was carried out to select texts for the experiment. Thirteen second-year optometry students read three texts each, filled in appropriate diagrams, completed their PoP, and took a test. Participants read each text only once and were not allowed to review previously read texts. This ensured that at least three participants read each text. The difficulty level of eight expository texts was comparable, as measured by the mean (± *SD*) percentage performance on questions about the causal relationship (75.41 ± 11.95%). To balance the variety of text types, we selected three texts that demonstrated serial causal relationships and the remaining three demonstrated both serial and simultaneous causal relationships.

### Prediction of performance (PoP)

The participants provided their PoP for their understanding of causal relationships for each text immediately after completing the diagram. The PoP was graded on a 6-point scale ranging from 0% (not at all confident) to 100% (fully confident), with the points on the scale corresponding to 0%, 20%, 40%, 60%, 80%, and 100%. When making their prediction, participants could see the text’s title on the screen, along with the question: How accurately do you believe you will be able to answer questions on the cause-and-effect relationship indicated in the text?

### Pre-study instructions

Prior to the study, the researcher instructed all participants to familiarize them with the steps and procedures. A video with a brief explanation of the study protocol, expository texts, cause-and-effect relationships, diagram completion, the PoPs, the post-test format and, the think-aloud method (including a demonstration of the think-aloud) was created and shared with the participants a day before the study. This allowed them to become familiar with the study, but more crucially, it guaranteed that the participants received consistent and neutral instructions. The data collection was initially planned in a one-on-one, face-to-face mode. The Covid-19 pandemic restricted face-to-face interaction, and the experimental procedure was shifted to a Zoom videoconferencing platform combined with the Qualtrics^**XM**^ survey software. Study workflow and procedures were carefully incorporated into the Qualtrics^**XM**^ survey software to assure procedural consistency across participants. Electronic consent from the participants was obtained at the end of the pre-study instructions.

### Procedure

**Fig. 1:**
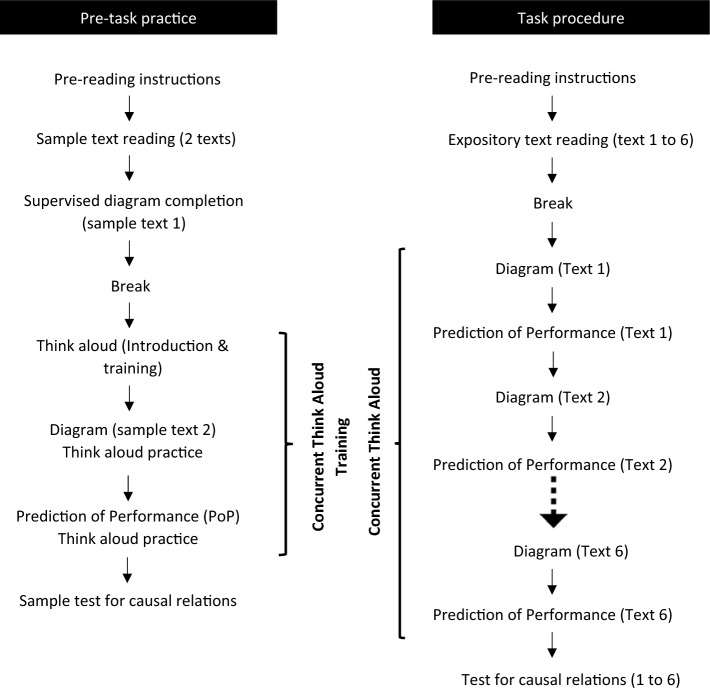
Overview of the pre-task and task procedures

On the day of the task, participants joined over Zoom. They were then asked to recall the important think-aloud instructions. Participants were then provided with a quick recap about the expository text, cause-effect relationships, diagram completion, think-aloud procedure, PoP, and the test at the end (Fig. 1). Next, they read two sample texts for practice: one with serial and the other with simultaneous causal relationships. Supported by the researcher, they completed the diagram for the first sample text. The researcher observed them thinking aloud while they were completing the second diagram and during the subsequent PoP. If they were silent for longer than five seconds, they were reminded to resume thinking aloud. Following this, the participants answered sample test questions on causal relationships. After this practice session, the participants were asked to take a five-minute break.

The researcher made sure participants were in a quiet environment before the task. Once they were clear about the study steps, they received an email link to access the original study tasks on Qualtrics^**XM**^. They had to read six texts one after another at their own pace, but they could not reread passages (Fig. 1). They were also instructed not to take any notes. They were allowed to keep their video and audio turned off during text reading. However, to keep track of their progress, they were required to notify the researcher whenever they progressed from one text to the next. After reading all six paragraphs, they took a 5 to 7 minute break before starting to complete diagrams in the next stage. The order of diagrams followed the order of texts they had read. After completing all six diagrams, participants predicted their performance in the test for all six texts on a visual analogue scale from 0 to 100%. After the PoP, they moved to the final step, where they answered the test questions. They concurrently verbalized their thoughts during diagram completion and PoP.

The researcher was available online to guide participants throughout the task. To ensure a comfortable and undisturbed environment, participants were instructed to find a quiet place, mute their video, and engage in natural think-aloud verbalizations. The entire experiment was audio-recorded using a screen recording software, and digital voice recorders served as backup. After the think-aloud phase, retrospective questioning was conducted to clarify any unclear data (Noushad et al., 2023).

### Diagram scoring

Each diagram consisted of five text boxes connected with arrow marks, and one of the boxes (either the first one or the last one) was already filled in for the participants (Appendix A & B). Responses in the diagram text boxes (cause or effect) were scored (Table 2), which is in line with the recommendations by van Loon et al. (2014) and van de Pol et al. (2020). The responses in the four filled-out text boxes in the completed diagrams were scored as ‘correct’ if a correct step in the causal chain was provided. If a box was filled out with only factual information, even if that information contained details from the reading text, it was scored as ‘incorrect’ because it did not represent a correct step in the causal chain. Furthermore, when the participants provided a response that did not come from the text, this was scored as a ‘commission error’. If no response was provided in a diagram textbox, this was scored as an ‘omission’ (Table 2).

**Table 2:** Criteria applied to score the text box contents in the completed diagrams

Scoring criteria	Description
Correct response	An idea point in the causal chain is stated accurately; min = 0, max = 4
Commission error	A text box response that did not come from the text; min = 0, max = 4
Incorrect response	A response describes detail from the text, but not an idea point in the causal chain; min = 0, max = 4
Omission error	The number of blank or question-marked boxes left without a response
Number of completed boxes	Number of boxes filled in the diagram irrespective of the content correctness; min = 0, max = 4
Number of correct relations	Correct arrangement of two idea points in the causal chain; min = 0, max = 4

### Test response scoring

The scoring criteria outlined by van Loon et al. (2014) and van de Pol et al. (2020) were used as the standard for evaluating test responses. The number of valid causal connections given in the test response received a score between 0 and 4 (0 = no correct causal relations, 4 = all correct causal relations). Emphasis was given to comprehension. Therefore, responses were also scored as correct if they indicated that the participants understood what was implied or meant with the original text content, that is, a response indicating gist understanding.

## Data analysis

### Participant’s monitoring accuracy

Relative monitoring accuracy is a measure of the within-participant correspondence between confidence and accuracy. This refers to the extent to which someone knows which texts are better understood relative to another (Maki et al., 2005). If one text had a higher confidence level than the other, the performance level in the criterion test is also anticipated to be higher for that text. The number of concordances (where the performance prediction and criterion test score move in the same direction) and discordances (where the performance prediction and criterion test score move in the opposite direction) are the basis of determining the relative monitoring accuracy. Therefore, relative monitoring accuracy does not reflect the magnitude of difference between the monitoring judgment and performance (i.e., the degree of judgement precision). On the other hand, absolute accuracy captures the absolute correspondence between the judgment and test performance. A higher absolute accuracy (i.e., difference closer to zero) implies that a learner is capable of predicting his/her performance accurately in a task or assessment, which may indicate the appropriate use of predictive cues during the process of self-monitoring. In real-world learning settings, having an accurate judgment of current level of knowledge and how and where that differs from what is expected (e.g., on the test) is a crucial aspect of metacognitive monitoring and essential to adequately regulate further learning. This aspect of metacognitive monitoring corresponds to absolute accuracy. Thus, research in the diagramming paradigm and metacognition more generally emphasize the significance of measuring absolute monitoring accuracy (De Bruin & van Gog, 2012; Pijeira-Díaz et al., 2023). Consequently, we decided to use absolute accuracy to categorize the participants as good and poor monitors.

### Categorizing good vs poor monitors

The purpose of this study is to identify the most diagnostic cues used by the participants. Hence, it is necessary to divide the participants into good and poor monitors. Based on their absolute accuracy scores, we divided the participants into three: the highest, the middle, and the lowest. This divided the total sample of 31 participants into the top one third (good monitors, *n* = 10) and the lower one third (poor monitors, *n* = 10), leaving 11 participants in the middle one third.

### Reflexive thematic analysis (RTA)

Think-aloud data in this study is a qualitative account of participants’ thought processes verbalized during the diagram completion and in the subsequent PoP. It was analyzed using reflexive thematic analysis (RTA) developed by Braun and Clarke (Braun & Clarke, 2006, 2019, 2021), underpinning a constructivist epistemological approach. This means that the patterns of cue use in the monitoring process were meaningfully constructed by the research team’s thoughtful interpretation of participants’ verbalizations. An ‘experiential’ qualitative data analysis approach was adopted to organize, prioritize, and interpret participants’ verbalizations. An overarching deductive framework was employed to ensure the initial data coding and theme construction were relevant to the research question. At the same time, an inductive approach was constantly shadowed to remain flexible and open to best represent the information presented by the participants. For instance, from the think-aloud data of participants, only those utterances suggestive of cue generation and utilization were considered for coding. Coding utilized both semantic and latent approaches. Semantic coding focuses on the descriptive surface meaning of what participants have communicated. In contrast, latent coding goes beyond the surface level of the data attempting to extract hidden meanings and assumptions (Byrne, 2022). Examples of semantic codes included ‘information processing cue’, ‘readability cue’, and ‘overall low confidence in content correctness’. Examples of latent codes included ‘cautious underestimation’ and ‘falsely overconfident’. For instance, “the overall impression of diagram completion” could be a feeling of ease or difficulty of comprehension. However, it may not always be a reliable comprehension cue unless accompanied by evidence of comprehension, such as a diagram that accurately represents the causal relationship. These relationships were thoroughly examined during the latent coding stage. If there was no correlation between the “feeling of comprehension” and the completed diagram, it was coded as “falsely overconfident”. Likewise, the memory retrieval cues (such as fluency, memorizability, quantity, and quality of retrieval) were analyzed together with the quality of the comprehension cue. For example, if a participant completed a diagram that was incorrect but said that s/he could easily recall the information, it was coded as an “incomplete diagram with the commission and incorrect responses.” However, if the memory retrieval cue was related to the accurate representation of the causal chain in the diagram, it was coded as “comprehension dominant PoP.” These approaches reflected the theoretical assumptions of analysis (constructive and interpretive) by giving due consideration to the semantic and latent meanings of the information provided by the participants. Coding of the transcribed data (both semantic and latent) was carried out manually using ATLAS.ti, a qualitative data analysis program. The program then enabled the comparison of cue use between good and poor monitors by generating a code document table and a Sankey diagram.

The data analysis process followed the six-phase analysis approach recommended by Braun and Clarke (2006). Phase one is to get familiarized with the entire data. One researcher (BN) manually transcribed all participants' think-aloud recordings verbatim and anonymized the transcripts. This allowed the researcher to familiarize himself with the whole dataset beforehand. In the second phase, the pieces of information relevant to the research question, such as cues suggestive of learning monitoring, were identified and coded. In the first part of this phase, only semantic coding was carried out using a broad coding framework to recognize various types of cues utilized by the participants (Table 3). The two main cue categories identified for semantic coding were comprehension-based cues and heuristic cues. The comprehension-based cues also included memory-retrieval cues (Griffin et al., 2009). The cues that arise from processing or constructing a specific text representation were termed as comprehension cues. All other cues that do not relate to the construction of a specific text representation were considered heuristic cues (referred to as surface cues in this study). This approach to classifying cues was adapted because the diagram completion intervention was designed to make comprehension cues more salient or available to learners (Prinz et al., 2020b). Semantic coding was followed by latent coding to identify the underlying patterns of cue use and relevant interpretations concerning the research question. In phase three, the coded data were reviewed, re-reviewed, analyzed, and combined according to their shared meaning to generate prospective themes. Several codes were combined and collated into similar underlying concepts or features. A few other codes were moved to the miscellaneous category as they did not fit within the overall analysis. In phase four, a review and reiteration of codes and initial themes were done to narrow down to the four final themes. These themes were named and defined in phase five. Finally, the report was written in phase six.

**Table 3:** The semantic coding framework developed using prior research as a reference to categorize the various types of cues employed by participants

Categories	Subcategories	Description
*Comprehension cues*		
Text comprehension	Situation level cue	Utterances that indicate situation-model comprehension
	Overall impression of the situation model (positive)	Utterances appear to be comprehension cues but convey the situation model's overall impression
	Overall low confidence in situation model (negative)	Responses indicating uncertainty regarding the situation model
Diagram comprehension	Overall impression of diagram completion	Expressions describing the overall impression of diagram completion
	Specific content correctness	Verbalizations on correctness of text box contents
	Causal relationship	The diagrams’ causal relations’ quality (correct relation or lack of relation)
	Omission cues	Verbalizations on missing details in the causal chain
	Overall low confidence in diagram correctness	Uncertain text box responses
*Memory retrieval cue*		
Memory retrieval Cue	Retrieval Fluency cues	Information’s ease of recall
	Memorizability cue	Whether one will (or will not) recall text information
	Retrieval Quantity cues	Amount of information accessed from the memory
	Retrieval quality cue	Overall quality of the memory retrieval
*Surface cues*		
Reading text surface cues	Reading fluency cues	How fluently (or less fluently) the text was read
	Information processing cue	How easily (or with difficulty) the information in the reading text was processed
	Readability cues	The ease (or difficulty) to understand the text
	Reading text length cues	Refers to the length of the texts
	Amount of textual information cues	The amount of information present in the text
	Vocabulary cues	Familiar or unfamiliar vocabulary in the text
	Reading time cues	Time taken to read the texts
	Factual information cues	Verbalizations on factual content from the text
	Reading text comparison cue	Comparing a reading text to another
Diagram surface cues	Diagram completion fluency cues	Ease (or difficulty) in completing the diagram
	Diagram completion time cues	Time taken to complete the diagram
	Diagram comparison cue	Comparing a diagram experience with other/s
Others	Prior knowledge cues	Previous knowledge about the text content
	Topic interest	Specific interest for a particular topic

Reflexive thematic analysis is considered a reflexive and interpretive account of researchers involved in analyzing qualitative data. This reflective and interpretive data analysis happens at the intersection of the dataset, theoretical assumptions underlying the analysis, and researchers' analytical skills and resources (Braun & Clarke, 2019). Following RTA's interpretive nature, the data analysis was primarily done by the first author (BN). The second (PWMVG) and third author (ABHdB) consistently contributed to sense-checking the ideas and exploring richer interpretations of the data. The researchers' regular team meetings facilitated this process.

## Results

### Monitoring accuracy

The relative monitoring accuracy in this study was observed to be *M* Gamma = 0.23 (*SD* = 0.57) following delayed diagram completion for the examination of causal relationships. This was estimated using the intra-individual *gamma* correlation between the participant’s PoP and their actual test score. Their overall absolute monitoring accuracy and for the higher, middle and lower one-third split categories are summarized in Table 4. Overall accuracy was estimated as the mean of the absolute accuracy scores of the 31 participants. The absolute monitoring accuracy (0.24) indicates a moderately higher score.

**Table 4:** Means (and standard deviations) of absolute monitoring accuracy of participants and their three split categories

	Mean (SD) (n = 31)	Higher 1/3rd (n = 10)	Middle 1/3rd (n = 11)	Lower 1/3rd (n = 10)
Absolute monitoring accuracy	0.24 (0.11)	0.14 (0.04)	0.23 (0.02)	0.34 (0.06)

### Good monitors vs poor monitors—diagram responses

Table 5 represents the diagram responses of the good and poor monitors. In the group of good monitors, the mean numbers of correct text box responses and correct causal relationships were significantly higher, whereas the poor monitors committed a greater number of commission errors (as determined by independent-samples *t* tests). That is, good monitors filled in a greater number of text boxes correctly, produced more causal connections, and committed fewer commission errors. The number of completed boxes seems to be a more diagnostic cue for students with high monitoring accuracy since they completed more boxes correctly. In contrast, students with low monitoring accuracy made more incorrect text-box completions (i.e., with commission errors of which they appeared unaware). The two groups did not differ in terms of omission errors (empty text boxes), and they appeared to have been utilized those cues without much difficulty.

**Table 5:** Means and standard deviations for number of completed boxes, correct responses, correct relations, omission errors, incorrect responses and *p* values for the contrast tested

	Good monitors		Poor monitors		*p*	*t*
	*M*	*SD*	*M*	*SD*		
Completed boxes	3.34	1.26	3.27	1.26	0.75	0.32
Correct responses	2.59	1.33	1.93	1.23	0.006*	2.81
Correct relations	2.12	1.46	1.40	1.40	0.007*	2.73
Omission errors	0.66	1.23	0.73	1.26	0.75	0.32
Commission errors	0.14	0.43	0.52	0.75	0.0009*	3.39
Incorrect responses	0.61	0.72	0.82	0.93	0.18	1.35

^*^Means differ significantly, *p* < 0.01

### Reflexive thematic analysis of think aloud data

**Fig. 2:**
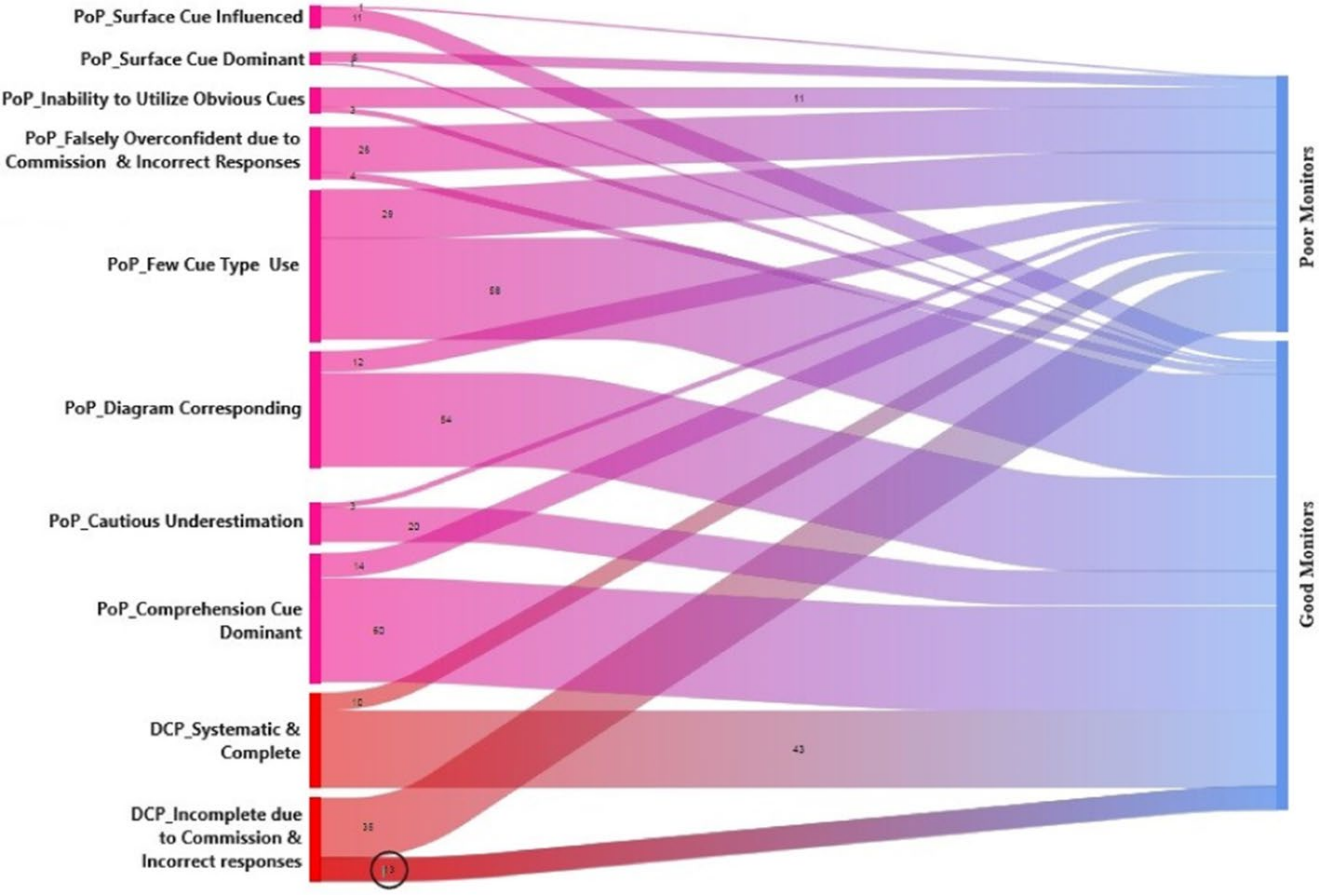
Sankey diagram depicting the pattern of cue use between good and poor monitors. The width of the diagram node is proportional to the quantity represented. (PoP-Prediction of Performance; DCP-Diagram Completion Process)

This study identified several noticeable differences in cue use patterns between good and poor monitors. The key distinction was the reliance on cues that reflect their situation model level, which is in line with the causal relationship described in the learning text. Good monitors tend to rely more on such cues when making their predictions (PoPs). The diagram completion exercise is known to help reflect their understanding of these causal relationships. However, the inaccuracies in completing the diagram can mislead poor monitors. Another characteristic observed was an underestimation tendency among good monitors and, the negative influence of nondiagnostic cues on the monitoring accuracy of both good and poor monitors. Each theme described below is accompanied by quotes that are illustrative, succinct and representative of the patterns in the data. A visual representation of the cue use pattern is shown in the Sankey diagram of ATLAS.ti. (Fig. 2).

### Theme 1—use of comprehension cues is the key to accurate monitoring

In this study, participants who predicted their performance based on comprehension-specific cues showed better accuracy in monitoring their performance (as indicated by the comprehension cue dominant node in Fig. 2). Completing diagrams act as self-tests to assess the situation-level model of comprehension. By filling in the text boxes in the diagram, participants would have been able to evaluate their comprehension level by reconstructing the causal relationship between idea units. *(S1_P3025_PoP6)*.

*The diagram is properly completed, and I remember the content clearly. What happens when the corneal tissue is reshaped, the corneal nerves will get damaged. So, it affects the communication between the cornea and the lacrimal gland, okay... it causes the lacrimal gland to produce less amount of tear, which then causes dryness... okay... 95%. (S1_P3025_PoP6).*

Good monitors' prediction of their performance was more aligned with their completed diagram, indicating the potential influence of the diagram completion exercise in guiding learners in monitoring (as shown in Fig. 2, the ‘diagram corresponding to PoP’ node). Additionally, the diagram completion process was systematic and complete among good monitors (represented by the ‘DCP-Systematic and complete’ node in Fig. 2), whereas poor monitors had incomplete diagrams due to commission errors and incorrect responses (‘indicated by the 'DCP-Incomplete due to commission and incorrect responses’ node in Fig. 2). Text comprehension is valid when the idea units of the causal chain are restored in an appropriate and logical manner. Similarly, failure to remember idea units in the causal chain may prompt students to scale down their predictions (S1_P3028_PoP4).

*As the age increases, some of the endothelial cells will die… (recollects the causal chain) …increasing the size or changing the shape. After that, there is one more thing that happens. Then, the water from the aqueous humor will enter the cornea, which causes corneal swelling. That is the reason for corneal oedema. But I don’t know what is happening in between. So... I will give 70%. Okay. (S1_P3028_PoP4).*

Several text and diagram-specific comprehension cues were accessible to participants throughout the diagram completion task. The ability to recall the causal connections was the most important of these cues, and it likely prompted participants to use cues indicating their level of confidence. (S1_P3031_PoP1).

*…when I was reading the paragraph, I know that the retinal pigment epithelium, which is the inner layer of the retina. And, also, how it supports the photoreceptor cell function. I know the relation between the age-related macular degeneration and when the person gets older, how it causes the photoreceptor cell death. And... how does the photoreceptor cell death happens too. So... I think...my level of confidence is 90 or 95? Am not sure. Okay...I will put it in between. So... between 90 & 95. Okay (S1_P3031_PoP1).*

Reconstructing the causal chain by filling out text boxes and gaining comprehension-specific information from that experience could be a valid source of predictive cues. These cues include overall confidence in the completed diagram (S1 P3004 PoP5), the accuracy of the text box contents, and the boxes that could not be filled out (omission of text boxes). In situations where the text boxes are unfilled or if participants couldn’t recall the idea units in one or more text boxes, monitoring decisions were easier and more accurate.

*…this one was easier ...I think I remembered most of the details and most of the essential information I needed to complete the diagram .... and I think it is well organized and easy to understand ...so am gonna put in the scale of .... let me see...btw 85 and 90. Yes. (S1_P3004_PoP5)*

*I will get myself...hmmm...25%...because I forgot more information...forgot more key words ...and, I don’t complete all the boxes in the diagram ...I will keep myself 25%. (S1_P3008_PoP2)*

The evidence, therefore, indicates the value of the diagram completion activity as a means of enabling learners to gain access to valid cues of situation-level comprehension.

### Theme 2—commission errors and incorrect responses in the diagram is associated with overconfidence (Poor interpretation of cue manifestation hinders monitoring accuracy)

The completion of the diagram's text boxes was found to serve as a valid cue for participants. However, occasionally, text boxes were filled with information outside the causal chain. If a box only contained factual information, even if it provided textual descriptions, the response was deemed incorrect because it did not reflect the correct steps in the causal chain. Similarly, if the response in the text box was different from the text, it was considered a commission error. Commission errors and incorrect responses suggest that the participant may have lacked true comprehension of the text at the situational level. However, their diagrams included a great deal of information that they believed to be accurate. This may result in an overestimation of learning judgments.

*...the paragraph was easy. I understand the content... like...the relationship of cause and effect. Also, I think I did a good job when I filled that information in the diagram. So…95%...Okay. Done. (Note: Of the four text boxes, three were filled with commission errors, while only one had the correct information. The participant scored only 25% on the test against his PoP of 95%) (S1_3006_PoP6).*

*I could remember all the information, and I completed the diagram fully …I will put it 100 here. Okay. (Note: all the text boxes were filled in with incorrect information and scored ‘zero’ on the test against his PoP of 100%) (S1_3024_PoP5).*

The above examples illustrate how inaccurate responses and commission errors can negatively impact the validity of monitoring judgments. Such mistakes in the diagram completion exercise can lead to overconfidence, resulting in poor monitoring accuracy. This trend was evident among poor monitors as illustrated in Fig. 2 (PoP-Falsely overconfident due to commission and incorrect responses node) and Table 5.

### Theme 3—tendency for cautious underestimation by good monitors

Good monitors tended to evaluate their performance as somewhat lower than their actual level of perceived performance. When asked about this discrepancy, it was noted that participants did not want to take risk and ending up scoring themselves higher.

*...this topic I could remember very well, so I will keep 80%. Because, I remember the information what I read from the paragraph...and...it is an easy topic. But still...I will keep it at 80. (S1_P3032_PoP5)*

*This diagram was not much of an issue. So, I will give 80. Because I could remember how drusen is formed, and the drusen slowly dysfunctions the RPE. This dysfunction of the RPE will weaken the photoreceptor cells and that causes their cell death. So, those are clear for me...I will give 80. Okay. (S1_P3028_PoP6)*

The two participants' excerpts above show their tendency to underrate their performance. They successfully completed the diagram, recreated the causal chain, and earned perfect scores on the test. However, they tended to underrate themselves. This caution was only exhibited by good monitors, especially when they dealt with unfamiliar topics or keywords.

### Theme—4: surface cues compromise the monitoring accuracy

If learners rely on cues aligned with characteristics relevant to the criterion test, their evaluations of learning are often accurate. However, surface cues do not indicate situation-level comprehension of a text. The time required to finish the diagram, the difficulty of reading the text, and difficult keywords or vocabulary are examples of surface cues that can affect learners' monitoring judgments. Such cues seemed to influence learners' monitoring judgments in this study.

*...I am going to keep 70%. I think…I took more time to remember about what to write in the boxes of the diagram. Also, there were a lot of information that I have read and I might forget some information. So...I will keep 70%. (S1_P3032_PoP4)*

In the above example, the participant used surface cues such as ‘the time required to complete the diagram’ and ‘the amount of textual information’ as their primary cue for predicting performance. It was evident that even experienced monitors were susceptible to surface cues. For instance, the following excerpt illustrates how the participant considered ‘prior knowledge and reading text comparison’ surface cues in addition to the comprehension cue. This influence of surface cue appears to have caused underestimation here (Fig. 2; PoP-Surface cue influenced node).

*...this was a new topic for me. I have understood the mechanism of how the silver halide in the photochromic lenses helps to block the UV rays. If I get questions based on that, I will not say fully confident. Because, I don’t have a detailed understanding of this topic since it is new for me. So, let me give 75. The highlighted portions in paragraph I clearly understand. Since I understood the paragraph content better than other paragraphs, I will keep it at 80. Should I go to 75? I shouldn’t go to 85. So...I will keep it in the middle...80%. Done. (S1_P3023_PoP6; this participant perfectly filled in the diagram and scored 100% in the test)*

Our data suggests that relying primarily on surface cues may lead to poor judgment accuracy. For instance, the participant below *(S1_P3006_PoP5)* relied primarily on the ‘readability cue’ and the ‘vocabulary cue’ to make a judgment, but the filled-in diagram contained incorrect responses and lacked causal connections. This participant received ‘zero’ score on the test, demonstrating a possible negative association with surface cues for monitoring (Fig. 2; ‘PoP-Surface cue dominant’ node).

*...when I read the paragraph, there was some new information. But, when I read it, I read it easily. But I don’t remember a word in it. Coz I haven’t come across this word before. So, this new information I couldn’t remember. And, when I completed the diagram, it was easy, but the only problem is I cannot remember that one word. So, I will keep... 80…coz of that word. (S1_P3006_PoP5)*

Based on our data, it seems that surface cues had a distracting effect on participants. This distraction can lead to a decrease in accuracy, especially when participants are overconfident due to commission errors or incorrect responses. Likewise, surface cues causes some underconfidence among good monitors.

Regarding cue use, poor monitors observed to have difficulty in utilizing obvious cues in predicting performance (as demonstrated by the node 'PoP-Inability to utilize obvious cues' in Fig. 2). For instance, despite realizing that they had poorly completed the diagram, they failed to consider such cues and rated themselves higher. Moreover, poor monitors considered multiple cue types than good monitors when making judgments (as indicated by the node ‘PoP-Few cue type use’ in Fig. 2).

## Discussion

This study explored the cognitive processes underlying the use of mental cues for performance monitoring between good and poor monitors following a cue-generation intervention. Those that relied on comprehension cues (e.g., the overall impression of the situation model cue, causal relationship cue, omission cue, correctness of textbox content cue, etc.) displayed better monitoring accuracy. On the other hand, it is likely that commission errors and incorrect responses made by participants during diagram completion have led them to become overconfident. The influence of surface cues and the usage of multiple cues was evident in reducing the monitoring accuracy of participants, particularly the poor monitors. The outcomes of this study provide new insights into the possible factors contributing to variations in monitoring accuracy.

These observations advance our understanding of the beneficial effects of a delayed diagram completion intervention. The primary purpose of this study is not to estimate the effect of diagram completion activity on the monitoring accuracy of learners (which has been established by (Van de Pol et al., 2019; van Loon et al., 2014)), but to gain a deeper understanding of the underlying mechanism of cue utilization. Previously, Thiede and colleagues attempted to explore the types of cue use by asking learners to self-report retrospectively after completing a delayed summary generation task (Thiede et al., 2010). Prior studies utilizing the diagram completion paradigm attempted to empirically estimate cue utilization and cue diagnosticity based on participant responses in the completed diagram (Van de Pol et al., 2019; van Loon et al., 2014). This study is the first attempt to obtain a direct account of the cognitive processes underlying cue utilization following a cue generation task using a think-aloud technique. Another contribution of this research is that it attempted to introduce the diagram completion exercise as an instructional strategy for learning complicated texts containing cause-effect relationships in a real-world setting of health professions education. The cue-utilization framework clearly proposes the possible influence of non-diagnostic cue use among poor monitors (Koriat, 1997; Thiede et al., 2010). Therefore, a further focus of this study was to shed light on the pattern of cue use among good and poor monitors of learning.

The two types of monitoring accuracy measures are independent since they focus on different aspects of metacomprehension skills and hence differ in their implications for educators and learners in real-world learning environments. While absolute accuracy provides feedback about learners' monitoring of their actual level of knowledge or skill and helps identify gaps, relative accuracy provides a comparative measure and feedback to learners on whether they can identify differences in knowledge and skill across tasks or topics. The bias index complements the absolute accuracy measure because it informs if learners are over- or underconfident, which could be a sign of dysfunctional cue use, such as reliance on surface cues (Gutierrez de Blume, 2022). Therefore, these accuracy measures help teachers tailor their instructional approaches and provide targeted and constructive feedback to learners on their self-monitoring skills. Teachers could incorporate monitoring accuracy measures more commonly in their teaching and analyse with students how and why they are (in) accurate (Sargeant et al., 2008). This would require explicit support from teachers to make this instruction effective, but if done effectively it would support the development of metacognitive skills (Medina et al., 2017).

The distinction between the good and poor monitors observed in this study was their extent of reliance on the situation-level comprehension cues to base their monitoring decisions on (Thiede et al., 2010). The causal connection between the idea units in the text and the reinforcement of the same when they completed the diagram were the two sources of cues. Good monitors relied on comprehension-specific cues for their judgement (text comprehension, diagram comprehension, or both) compared to poor monitors. Further, the judgements of good monitors were congruent with the completion and correctness of the diagram, indicating that the good monitors may have accurately identified and utilised the situation-model cues from diagram completion (Thiede et al., 2003; Thiede et al., 2010; Van de Pol et al., 2019; van Loon et al., 2014). The diagram completion task acts as a self-test for memory retrieval, providing learners with cues representative of their actual comprehension. The utilization of cues is always dependent on the availability of cues. Those with a higher level of comprehension have access to comprehension-specific cues, but those with a lower level of comprehension do not. It suggests that identifying and interpreting the validity of available cues during monitoring requires additional assistance, particularly for those with impaired comprehension (Thiede et al., 2010; van de Pol et al., 2020).

Poor monitors frequently filled in the diagrams with information from outside the causal chain, that is, with incorrect responses and commission errors. That probably prompted them to base their judgement on an incorrect cue, i.e., the successful completion of the diagram. It suggests that the poor monitors failed to recognise the incorrect responses and commission errors they had incurred while completing the diagram (van de Pol et al., 2020). As indicated previously, such inaccuracies could have prompted the learners to score themselves higher (overconfidence) and compromised the monitoring accuracy (Dunlosky & Rawson, 2012; Dunlosky et al., 2005; Finn & Metcalfe, 2014). In another sense, the mere use of a diagnostic cue, such as ‘*successful completion of the diagram’*, does not ensure accurate monitoring, unless it accurately reflects situation-level comprehension. Poorly completed diagrams with commission errors or incorrect responses, can compromise the quality of the situation-model (van de Pol et al., 2020). In the current study, this may have led to a discrepancy between the completed diagram and the performance prediction made by the poor monitors. This underscores the significance of accurately interpreting available cues and effectively utilizing them in the judgement process. Good monitors, in contrast, made fewer commission errors and incorrect responses. Van de Pol and colleagues described the term ‘cue manifestation’ as the ability of learners to accurately judge the right interpretation of the cues available for them (e.g., correct causal relations, presence of commission errors, the relevance of omission errors, etc.) (van de Pol et al., 2020). Therefore, monitoring accuracy requires an ability to recognize diagnostic cues, properly infer them and appropriately utilize them to base their judgement. In general, the poor monitors struggled in this area compared to the good monitors.

Good monitors exhibited a tendency to judge themselves a bit lower than their actually perceived performance. This pattern corresponds to the trend observed in the literature, where high and low performers are reported to show biases in their self-judgement, but in different degrees in the absolute scores. Low performers are less accurate in their judgements as a result of a significant overestimation of their own performance. On the other hand, high performers are more accurate in their judgement and in general, underestimate themselves (Dunning et al., 2003; Foster et al., 2017; Saenz et al., 2017). Good performers are known to have better metacognitive awareness compared to poor performers. At times, their metacognitive awareness might not be convincingly enough to judge them correct but, prompting them to lower their level of confidence (Tirso et al., 2019).

Several surface cues relevant to the text and diagram completion task were used by the participants in this study (Table 3). It is evident from the findings that the use of such surface cues had a detrimental impact on the monitoring accuracy of both poor and good monitors, especially the poor monitors. It suggests that a greater dependence on surface cues could result in a decline of monitoring accuracy. This reconfirms the findings of Thiede and colleagues, who observed that those participants who reported using only surface cues had significantly lower gamma values (− 0.03) than those who reported using comprehension-based cues (0.71). In addition, they found that monitoring accuracy declines when participants report using a combination of surface cues and comprehension cues (− 0.33) (Thiede et al., 2010). It also supports an observation drawn from this study where poor monitors relied on multiple cues than good monitors who utilized fewer comprehension-specific cues for performance monitoring. Consideration of multiple cues for monitoring decisions by poor monitors indicates their inability to recognize valid cues, which would further complicate their judgment process. When people attempt to make predictions on the basis of imperfect probabilistic cues, they may be unknowingly biased by their accessible mental representation, which does not necessarily represent the essential information to reach a conclusion (Newell & Shanks, 2014). This emphasizes the need to address the effect of surface cues and the usage of multiple cues when devising interventions to improve monitoring accuracy.

In this work, we observed a lower relative monitoring accuracy (*M* gamma = 0.23) compared to previous diagram completion studies. This may not qualify as a direct comparison with previous diagram completion research due to the difference in primary research focus, incomparable sample size, and lack of a control group. However, it is worth noting that the unproductive effects of commission errors, incorrect responses, surface cues, and multiple cue use differentiated poor monitors from good monitors, suggesting an inter-individual difference in cue-diagnosticity (van de Pol et al., 2020). According to the cue-utilization framework, those cues that are most diagnostic of students’ text comprehension should be used and non-diagnostic cues should be ignored (Koriat, 1997). Although the diagram completion approach generates valid and diagnostic cues, it can also misguide learners if the diagram is completed using incorrect information. Ultimately, it is the learner's responsibility to accurately interpret and utilize these cues. That remains a challenge, especially for poor performers. Therefore, future interventions could focus on strategies that provide feedback to make students aware of their errors, so as to improve their monitoring accuracy (van de Pol et al., 2020).

The observations from this study have considerable implications for the self-monitoring of health professionals regarding their professional development. Medical trainees who engaged in self-monitoring achieved higher overall performance levels (Jamshidi et al., 2009; Netter et al., 2021), made more performance gains (Halim et al., 2021), showed a positive impact on patient outcomes (Holmboe et al., 2005), and demonstrated improved workplace behaviour (Hale et al., 2016) compared to those trainees who did not engage in self-monitoring. In the medical sciences, self-monitoring is often tested by measuring the accuracy of performance self-judgement in comparison to an expert evaluation or standardized clinical protocols, which have shown varied levels of agreement (Casswell et al., 2016; Moorthy et al., 2006; Quick et al., 2017). In general, novice trainees displayed less accuracy in self-monitoring than their experienced senior trainees or faculty experts (Casswell et al., 2016; de Blacam et al., 2012; Moorthy et al., 2006). These observations have incited the development of interventions to support novices with peer feedback (Evans et al., 2007), expert coaching (Wouda & van de Wiel, 2014), simulated self-training tools (Veaudor et al., 2018), and self-assessment instruments (Andersen et al., 2020). While these studies have shown better agreement in performance monitoring, they lack a deeper understanding of the factors that compromise the monitoring accuracy of learners, as well as the inter-individual and task-specific variations that could guide researchers to construct customized feedback or feedforward interventions to improve monitoring accuracy. In that regard, the observations from the present study provide an essential contribution.

While attempting to draw conclusions from this study, it is necessary to account for some shortcomings. We lacked a thorough understanding of the cue use patterns of learners without a control condition, which would have allowed us to better comprehend the effects of the diagram completion intervention. Nevertheless, the current study has revealed areas where future research should focus on to enhance the monitoring accuracy of learners. Second, cues used for the semantic coding of transcripts have been reported in the literature; however, analyzing the impact of individual cues on monitoring accuracy in the context of diagram completion was not within the scope of this study. It would be valuable for future research to investigate the predictive validity of these cues in authentic learning contexts. Third, we may debate the effectiveness of the think-aloud method for identifying concurrent cognitive processes. Reactivity (the higher cognitive load involved with reporting and thinking simultaneously) and non-veridicality (the non-concordance between actual thought processes and verbalizations) are the two key criticisms of this method. The key to avoiding reactivity and non-veridicality is to provide participants with thorough instructions and training prior to think-aloud. We have attempted to address these issues adequately.

## Conclusion

This study aimed to expand the present understanding of the potential sources of errors that learners may make when monitoring their own performances, despite accurate prompts leading to reliable cues. As demonstrated here, the use of cues that reflect the situation-level mental text representation would assist learners in achieving greater monitoring accuracy. Equally significant is guiding learners away from the wrong interpretation of cues that result from commission errors and incorrect responses. The influence of surface cues is an additional obstacle to achieving reliable monitoring. These observations emphasize the importance of supporting learners to select and use diagnostic cues while monitoring their performance.

## Appendix A

### Text “Photochromic Lenses & UV Protection’’

Light is crucial for vision. It gets reflected from objects and enters to the eye so that we can see them. Natural light or sunlight includes harmful radiations such as Ultraviolet (UV) radiations. One of the best options to protect eyes from these harmful radiations is by using photochromic lenses; (*Photo* refers to light and *Chromic* refers to color). These lenses turn darker when we go out in the sun and becomes a clear lens when we move to indoors. In the sunlight, the exposure to ultraviolet rays makes the lens darker and when we move away from the UV rays, it returns back to its original state. Photochromic lenses are slightly different in their structure compared to regular lenses. Tiny molecules of silver halide are embedded within the photochromic lens which is invisible and clear until exposed to UV rays. Upon exposure, the photochromic molecules (silver halide) breaks into silver and halide. This process causes the silver ions to transform into elemental silver. Elemental silver then absorbs the ultraviolet rays and changes into darker color. Since the elemental silver absorbs UV rays, those harmful rays will not enter the eye and cause damage.

## Appendix B

### Text “Endothelial Cell loss & Corneal Edema’’

The Cornea is a curved, transparent structure seen at the front of the eye. It has 5 layers. The innermost layer is the endothelial cell layer which is in contact with the aqueous humor. Aqueous humor is a clear, watery fluid seen between the cornea and the iris. The endothelium is a single layered structure made up of mostly six sided (hexagonal) cells. The junctions between these cells are tight. However, it allows nutrients from the aqueous humor to pass through the endothelium to reach the other layers of the cornea. But, water content from the aqueous cannot pass through these tight junctions. This restriction for water to enter the cornea is very essential to keep the cornea transparent. It is also important to note that, the endothelial cells do not divide or reproduce. If they are lost or damaged, new cells cannot be formed. As we get older, the number of endothelial cells decreases due to cell death. Since new cells cannot be formed, the nearby endothelial cells try to fill the gap left by dead cells by either changing their shape (known as ‘pleomorphism’) or by increasing their size (known as ‘polymegathism’). These changes in endothelial cells disturbs the tight junctions between them. Once the junctions between the cells are not tight enough, it allows water from the aqueous humor to enter the cornea. This additional water that reach the cornea makes the cornea swell, which is known as Corneal Edema.
